# Robotic versus laparoscopic ileal pouch-anal anastomosis for ulcerative colitis: an analysis of the Nationwide readmission database, 2016–2020

**DOI:** 10.1007/s10151-025-03285-2

**Published:** 2026-05-12

**Authors:** Ming-Hung Lee, Yu-Yao Chang

**Affiliations:** 1 Division of Colon and Rectal Surgery, Department of Surgery, Kaohsiung Veterans General Hospital, Kaohsiung, Taiwan; 2 Division of Colon and Rectal Surgery, Department of Surgery, Changhua Christian Hospital, No 135, Nanxiao Street, 50006 Changhua, Taiwan; 3 Department of Post-Baccalaureate Medicine, College of Medicine, National Chung Hsing University, Taichung, Taiwan; 4 Department of Nutrition and Health Sciences, Chang Jung Christian University, Tainan, Taiwan

**Keywords:** Ileal pouch-anal anastomosis (IPAA), Laparoscopic surgery, Nationwide Readmission Database (NRD), Robotic-assisted surgery, Ulcerative colitis

## Abstract

**Background:**

Minimally invasive ileal pouch-anal anastomosis (IPAA), including laparoscopic and robotic-assisted approaches, has been shown to improve outcomes for ulcerative colitis (UC), but few studies have compared outcomes between robotic-assisted and conventional laparoscopic IPAA. This study aimed to evaluate short-term outcomes of robotic-assisted versus laparoscopic IPAA.

**Methods:**

This retrospective cohort study utilized the U.S. Nationwide Readmission Database from 2016 to 2020. Patients aged ≥ 18 years undergoing robotic or laparoscopic IPAA for UC were included. Outcomes measured were in-hospital mortality, length of stay (LOS), hospital costs, complications, and 30-day and 90-day readmission rates. Propensity score matching (PSM) was employed to balance baseline characteristics, and multivariable regression was applied for adjusted analyses.

**Results:**

Among 820 patients with UC undergoing IPAA, 242 robot-assisted and 242 laparoscopic cases were matched after PSM. Baseline demographic and clinical characteristics were well balanced following PSM. Robotic-assisted IPAA was associated with higher total hospital costs compared with the laparoscopic approach (adjusted mean difference, 24,460 USD; 95% CI 7.69–41.24; *p* = 0.004). No significant differences were observed between groups in LOS, overall postoperative complications, or 30- and 90-day readmission rates.

**Conclusion:**

In this nationwide analysis, robotic-assisted IPAA was associated with higher hospital costs but comparable short-term clinical outcomes relative to laparoscopy. These findings suggest that while robotic IPAA is feasible and safe, its higher cost without clear short-term clinical benefit warrants careful consideration when selecting the surgical approach.

**Supplementary Information:**

The online version contains supplementary material available at 10.1007/s10151-025-03285-2.

## Introduction

Ulcerative colitis (UC) is a chronic inflammatory bowel disease (IBD) marked by persistent inflammation of the colonic mucosa [1]. Symptoms include diarrhea, abdominal pain, rectal bleeding, and tenesmus. UC is associated with systemic complications such as malnutrition and an elevated risk of colorectal cancer. UC incidence is rising globally, particularly in North America and Europe [2]. Peak onset occurs between ages 15 and 30 years, with a secondary peak in later adulthood [3]. UC treatment aims to induce and maintain remission, alleviate symptoms, and prevent complications [4]. However, around 20–30% of patients require surgery due to refractory disease, severe complications, or cancer risk [5].

Total proctocolectomy with ileal pouch-anal anastomosis (IPAA) is the gold-standard surgical procedure for UC [6]. IPAA restores intestinal continuity, preserves continence, and reduces cancer risk. IPAA, initially performed as an open procedure, has evolved with minimally invasive techniques, including laparoscopic, robotic-assisted, and transanal laparoscopic approaches [6, 7]. Laparoscopic IPAA offers advantages such as reduced postoperative pain, shorter hospital stays, and quicker recovery [8]. However, it is challenging due to the confined pelvic space and the need for precise dissection and suturing, especially in obese patients or those with prior abdominal surgeries [8].

Robotic-assisted surgery provides enhanced visualization, greater dexterity, and improved surgeon control, addressing the limitations of laparoscopy [9]. These features may improve outcomes in complex cases, such as those with narrow pelvic anatomy or extensive adhesions. However, robotic-assisted surgery involves longer operation times and higher costs, raising questions about its comparative benefit over laparoscopic IPAA.

While several single-center studies have examined this issue [10–12], the increasing use of robotic-assisted IPAA calls for a comprehensive evaluation of its safety and efficacy in real-world clinical settings. This study aimed to compare the short-term outcomes of laparoscopic and robotic-assisted IPAA using a large national dataset, providing evidence to inform surgical decision-making in patients with UC.

## Methods

### Data source and study design

This retrospective population-based cohort study extracted all data from the United States (US) Nationwide Readmission Database (NRD), a publicly available, all-payer database developed by the Agency for Healthcare Research and Quality (AHRQ) as part of the Healthcare Cost and Utilization Project (HCUP), a division of the US Department of Health and Human Services (DHHS). The NRD is sourced from the HCUP State Inpatient Databases and offers an accurate representation of total US hospitalizations and readmissions, regardless of insurance provider. It includes verified patient linkage numbers, enabling the tracking of individuals across hospitals within a given year while maintaining strict adherence to privacy guidelines. The NRD covers a full calendar year of data, with diagnoses and procedures recorded using the International Classification of Diseases, Tenth Revision, Clinical Modification (ICD-10-CM), and procedure codes (ICD-10-PCS) starting from the 2016 data year. The overview of the structure of NRD can be found at: https://hcup-us.ahrq.gov/nrdoverview.jsp.

### Study population

This study included patients aged ≥ 18 years who were admitted with a principal diagnosis of UC. Only patients who received IPAA either through a robotic-assisted or laparoscopic approach were included. Since the NRD only allows tracking of readmissions within the same calendar year as the index admission, patients admitted between 1 January and 30 September of each year from 2016 to 2020 were included to ensure at least 90 days of follow-up. Patients who had colon cancer or lacked information on age, sex, sample weights, or main outcomes of interest were excluded. The specific ICD-10 codes used are provided in Supplementary Table S1.

### Follow-up

After the index admission, patients were considered “at-risk” for hospitalization and were included in the follow-up period until 31 December of the admission year or until death.

### Ethics statement

This study used secondary, anonymized data, with no direct patient involvement. The use of deidentified data complies with ethical research standards and aligns with data protection regulations, as it was originally collected for purposes other than this specific study.

### Outcome measures

The primary outcomes measured included in-hospital mortality, length of stay (LOS), total hospital costs, and postoperative complications (during the index admission), and 30-day and 90-day unplanned (emergent) readmissions. Complications reported across all included patients were pneumonia, sepsis, urinary tract infection (UTI), surgical site infection, venous thromboembolism (VTE), respiratory failure/mechanical ventilation, acute kidney injury, shock, postprocedural intestinal obstruction, pouchitis, fistula, or mechanical issues with the pouch, other unspecified postprocedural complications of the digestive system, and “any complication.” These conditions were confirmed through the corresponding ICD codes, as summarized in Supplementary Table S1.

### Covariates

Covariates included age categories (groups within specific age ranges), sex, smoking, Charlson comorbidity index, insurance status (primary payer), weekend admission, year of admission, and major comorbidities. Comorbidities studied were polyps of the colon, obesity, diabetes, chronic pulmonary disease, chronic kidney disease, rheumatic disease, hypertension, ischemic heart disease/ heart failure, malnutrition, anemia, and use of immunosuppressive agents. These conditions were defined by relevant ICD codes documented in Supplementary Table S1. Additionally, hospital characteristics provided in the NRD, including bed size and hospital location/teaching status, were also included as covariates.

### Statistical analysis

Descriptive statistics were used to summarize patient demographics and clinical characteristics, with categorical variables presented as counts and weighted percentages and continuous variables as means ± standard error (SE). Group comparisons for categorical variables were conducted using the Rao–Scott chi-squared test, while weighted mean differences for continuous variables were analyzed using survey methods that account for stratification, clustering, and sampling weights, ensuring valid and robust statistical inferences within the context of complex survey designs. To minimize selection bias, 1:1 propensity score matching (PSM) was conducted on the basis of variables with *p*-values < 0.05 or standardized mean difference (SMD) ≥ 0.1 in the univariate analysis [13]. The matching process followed a one-to-many approach [14]. The method prioritizes “best” matches first and then proceeds with “next-best” matches until no more can be made. A two-sided *p*-value of < 0.05 was established as statistical significance. All analyses represented the complex survey design of the NRD, designed to ensure accurate national estimates. Statistical analyses were conducted using SAS version 9.4 (SAS Institute Inc., Cary, NC, USA).

## Results

### Patient selection

**Fig. 1:**
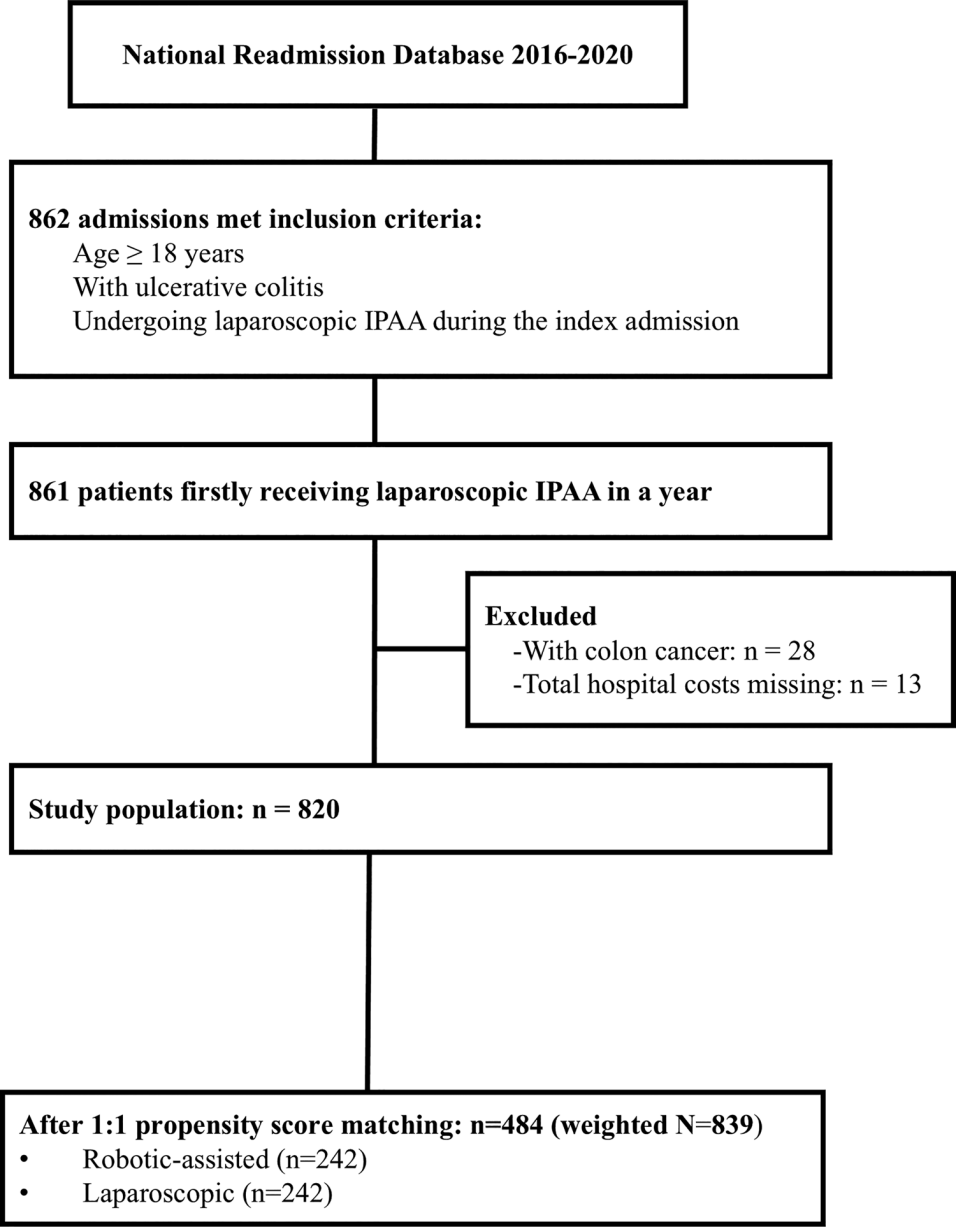
Flow diagram of the patient selection process

The selection process for the study population is shown in Fig. 1. A total of 861 patients with UC who underwent either robot-assisted or laparoscopic IPAA during the index admission were identified from the NRD between 2016 and 2020. After excluding 41 patients with colon cancer or missing data on total hospital costs, the final analytic sample included 820 patients. PSM was performed using variables with a *p*-value < 0.05, as presented in Supplementary Table 2. A total of 484 patients were selected in a 1:1 ratio, including 242 who received robot-assisted IPAA and 242 who received laparoscopic IPAA. After applying appropriate weighting, this matched cohort represents an estimated 839 hospitalizations across the USA (Fig. 1).

### Characteristics of the study population

Supplemental Table 2 summarizes patients’ demographic and clinical characteristics and outcomes before matching. The mean age of the patients was 39 years, and 57% were male. Significant differences in sex, smoking, anemia, year of admission, and hospital bed numbers were observed between the two groups—robot-assisted and conventional laparoscopic IPAA (Supplementary Table S2).

After PSM, the mean age was 39 years, and 62% were male. Baseline characteristics were well balanced between the two groups (Table 1). Patients who underwent robotic-assisted IPAA still had significantly higher total hospital costs compared with those who underwent laparoscopic IPAA (125,500 USD versus 100,400 USD, *p* = 0.004) and surgical complications (11.1% versus 6.4%, *p* = 0.047). No significant differences were observed in other outcomes, including medical or surgical complications, LOS, or 30-day and 90-day readmission rates. (Table 2).

**Table 1:** Characteristics of the study population (after matching)

Characteristics	All patients	Robotic-assisted		*p-Value*	*SMD*
	(*n* = 484)	Yes	No		
		(*n* = 242)	(*n* = 242)		
**Age, years**	38.8 ± 0.6	38.9 ± 0.9	38.7 ± 0.9	0.905	0.036
18–29	165 (33.9)	84 (33.6)	81 (34.2)	0.994	0.041
30–39	101 (21.0)	51 (21.2)	50 (20.8)		
40–49	98 (20.2)	48 (20.1)	50 (20.3)		
50–59	72 (14.9)	37 (15.5)	35 (14.3)		
60 +	48 (10.0)	22 (9.6)	26 (10.3)		
Sex				0.901	0.012
Male	304 (61.7)	155 (62.0)	149 (61.5)		
Female	180 (38.3)	87 (38.0)	93 (38.5)		
Procedure				0.780	0.026
Proctectomy	398 (81.0)	197 (80.5)	201 (81.5)		
Proctocolectomy	86 (19.0)	45 (19.5)	41 (18.5)		
Smoking	128 (26.5)	64 (27.0)	64 (26.0)	0.792	0.023
Insurance status/primary payer				0.211	**0.159**
Medicare/Medicaid	100 (19.6)	53 (19.4)	47 (19.8)		
Private including HMO	363 (76.4)	174 (75.1)	189 (77.7)		
Self-pay/no-charge/other	20 (4.0)	14 (5.5)	6 (2.4)		
Missing	1	1	0		
Major comorbidities					
Polyp of the colon	3 (0.5)	2 (0.6)	1 (0.5)	0.879	0.013
Obesity	40 (9.3)	21 (9.7)	19 (8.8)	0.721	0.033
Diabetes	32 (7.3)	15 (6.9)	17 (7.7)	0.773	0.032
Chronic pulmonary disease	45 (9.4)	19 (7.8)	26 (11.0)	0.252	**0.109**
Chronic kidney disease	9 (1.9)	4 (1.8)	5 (2.0)	0.853	0.018
Rheumatic disease	8 (1.6)	5 (2.2)	3 (1.0)	0.262	0.097
Hypertension	76 (15.8)	35 (14.4)	41 (17.2)	0.436	0.074
Ischemic heart disease/	5 (1.7)	2 (1.5)	3 (1.9)	0.851	0.030
Heart failure					
Malnutrition	38 (7.7)	19 (8.2)	19 (7.3)	0.757	0.032
Anemia	92 (19.8)	47 (20.8)	45 (18.8)	0.574	0.050
Use of immunosuppressive agents	37 (8.0)	15 (7.0)	22 (9.0)	0.445	0.072
CCI				0.933	0.068
0	380 (78.1)	192 (79.0)	188 (77.3)		
1	68 (13.5)	32 (12.5)	36 (14.5)		
2	19 (4.6)	10 (4.9)	9 (4.2)		
3 +	17 (3.8)	8 (3.7)	9 (4.0)		
Admission type				0.313	0.085
Elective	11 (2.0)	7 (2.6)	4 (1.4)		
Emergent	473 (98.0)	235 (97.4)	238 (98.6)		
Weekend admission	2 (0.4)	2 (0.8)	0 (0.0)	–	–
Year of admission				0.941	**0.114**
2016	67 (14.7)	31 (14.9)	36 (14.5)		
2017	96 (19.2)	47 (19.3)	49 (19.0)		
2018	104 (21.0)	57 (22.2)	47 (19.9)		
2019	106 (21.5)	54 (22.4)	52 (20.7)		
2020	111 (23.5)	53 (21.2)	58 (25.8)		
Hospital bed numbers				0.764	0.066
Small	22 (5.6)	11 (4.8)	11 (6.3)		
Medium	123 (25.0)	61 (25.5)	62 (24.6)		
Large	339 (69.4)	170 (69.7)	169 (69.1)		

Continuous variables are presented as mean ± SE; categorical variables are presented as unweighted counts (weighted percentage)

*CCI* Charlson comorbidity index

*p*-Values < 0.05 are shown in bold

**Table 2:** Comparisons of in-hospital outcomes between patients treated with and without robotic assistance (after matching)

Outcomes	All patients	Robotic-assisted		*p-Value*
	(*n* = 484)	Yes (*n* = 242)	No (*n* = 242)	
In-hospital mortality	1 (0.2)	0 (0.0)	1 (0.2)	**–**
LOS^a^	5.8 ± 0.3	6.0 ± 0.4	5.6 ± 0.3	0.424
Total hospital costs^a,b^	112.9 ± 4.5	125.5 ± 5.8	100.4 ± 6.4	**0.004**
Complications, any	74 (14.6)	42 (16.3)	32 (13.0)	0.285
Surgical complications	45 (8.7)	28 (11.1)	17 (6.4)	**0.047**
Surgical site infection	6 (1.0)	5 (1.8)	1 (0.3)	0.077
Pouchitis, fistula, or mechanical issues with the pouch	20 (4.3)	9 (4.4)	11 (4.3)	0.941
Other unspecified postprocedural complications of the digestive system	13 (2.3)	8 (2.8)	5 (1.8)	0.427
Postprocedural intestinal obstruction	6 (1.1)	4 (1.4)	2 (0.8)	0.557
Hemorrhage/hematoma/seroma	6 (1.1)	5 (1.7)	1 (0.4)	0.143
**Medical complications**	44 (9.0)	22 (8.6)	22 (9.4)	0.767
Pneumonia	3 (0.6)	2 (0.7)	1 (0.4)	0.683
Sepsis	11 (2.2)	5 (1.8)	6 (2.5)	0.561
UTI	9 (2.1)	4 (1.7)	5 (2.5)	0.531
VTE	13 (2.7)	7 (3.1)	6 (2.4)	0.651
Respiratory failure/mechanical ventilation	4 (0.7)	2 (0.7)	2 (0.7)	1.000
AKI	13 (3.0)	5 (2.2)	8 (3.8)	0.369
Shock	5 (1.1)	0 (0.0)	5 (2.2)	-
**30-day unplanned readmission**^**a**^	110 (22.6)	49 (20.8)	61 (24.4)	0.342
**90-day unplanned readmission**^**a**^	157 (31.9)	71 (29.3)	86 (34.6)	0.205

Continuous variables are presented as mean ± SE; categorical variables are presented as unweighted counts (weighted percentage)

*LOS* length of stay, *UTI* urinary tract infection, *VTE* venous thromboembolism, *AKI* acute kidney injury

*p*-Values < 0.05 are shown in bold

^a^Excluding patients who died in hospitals

^b^Thousand USD

### Associations between robotic surgery and outcomes stratified by procedure type

Adjusting for covariates with an standardized mean difference (SMD) ≥ 0.1 or *p*-value < 0.05 after PSM in the multivariable analysis, patients who underwent robotic-assisted IPAA had significantly higher total hospital costs compared with those who underwent laparoscopic IPAA (24,460 USD greater, 95% CI 7.69–41.24, *p* = 0.004). However, no significant association was observed between robotic surgery and other outcomes (Table 3).

**Table 3:** Comparisons of in-hospital outcomes between patients treated with and without robotic assistance

Outcome	Event numbers	Robotic-assisted	aOR/aBeta (95% CI)	*p*-Value
LOS^a^	**–**	Yes versus no	0.23 (−0.68, 1.13)	0.621
Total hospital costs^a,b^	**–**	Yes versus no	24.46 (7.69, 41.24)	**0.004**
Complications, any	74	Yes versus no	1.31 (0.80, 2.14)	0.285
Surgical complications	45	Yes versus no	1.82 (0.997, 3.33)	0.051
Pouchitis, fistula, or mechanical issues with the pouch	20	Yes versus no	1.01 (0.47, 2.18)	0.983
Other unspecified postprocedural complications of the digestive system	13	Yes versus no	1.62 (0.50, 5.23)	0.418
Medical complications	44	Yes versus no	0.90 (0.46, 1.74)	0.742
Sepsis	11	Yes versus no	0.72 (0.21, 2.49)	0.602
UTI	9	Yes versus no	0.73 (0.21, 2.52)	0.613
VTE	13	Yes versus no	1.25 (0.40, 3.89)	0.699
AKI	13	Yes versus no	0.60 (0.18, 2.01)	0.402
**30-day unplanned readmission**^**a**^	110	Yes versus no	0.79 (0.52, 1.21)	0.283
**90-day unplanned readmission**^**a**^	157	Yes versus no	0.77 (0.53, 1.13)	0.182

The multivariable models were adjusted for covariates with an SMD ≥ 0.1 or *p*-value < 0.05 after PSM, including insurance status/primary payer, chronic pulmonary disease,

and year of admission. Variables that could not predict the outcome (e.g., those with an empty set) were excluded from the model

Continuous variables are presented as mean ± SE; categorical variables are presented as unweighted counts (weighted percentage)

*LOS* length of stay, *UTI* urinary tract infection, *VTE* venous thromboembolism, *AKI* acute kidney injury

*p*-Values < 0.05 are shown in bold. SMD ≥ 0.1 are shown in bold

^a^Excluding patients who died in hospitals

^b^Thousand USD

## Discussion

In this nationwide cohort of patients with UC undergoing IPAA, robotic-assisted surgery was associated with significantly higher hospital costs compared to laparoscopic surgery. However, no significant differences were observed between the two approaches in short-term outcomes, including postoperative complications, VTE, LOS, or 30- and 90-day readmission rates. These findings, as did previous studies [9–11, 15, 16], suggest that although robotic-assisted IPAA may offer technical advantages in complex cases, these benefits may not appear to translate into improved short-term clinical outcomes, while incurring higher costs.

In the adjusted analysis, robotic-assisted surgery was not associated with an increased risk of postoperative VTE. The finding aligns with the recent multicenter study by Violante et al. [17], which also reported no significant difference in VTE incidence between laparoscopic and robotic IPAA. The slightly higher crude VTE rates among robotic cases may instead reflect residual confounding, including unmeasured variability in VTE prophylaxis protocols, institutional practices, or case complexity, rather than the surgical platform itself. Although longer operative time is a known risk factor for VTE in major surgery [18], the present nationwide data do not support a differential risk between robotic and laparoscopic IPAA after adjustment.

Several single-center studies have compared robotic-assisted versus laparoscopic IPAA [9–11, 15, 16], generally reporting comparable short-term outcomes despite longer operative times and higher costs for robotic procedures. Some previous single-center studies specifically evaluated outcomes such as intraoperative blood loss and functional outcomes at follow-up, which we could not assess due to the lack of related data in the NRD. A fairly recent review study looking at reports up to 2021 [15] noted that the robotic platform was safe to use for IPAA procedures, but that only minimal evidence supported its clinical advantages. A more recent review of studies through 2024 [16] concluded that the robotic approach was still in its initial stages for pelvic surgeries, and though it can be safely employed due to improved dexterity and visibility, data are still lacking on which to base surgical decisions. Other investigators evaluated the robotic approach to proctectomy and IPAA and found that it offered significant technical advantages to the laparoscopic approach, expanding minimally invasive surgical techniques to IPAA [9]. Gebhardt et al. [10] in 2022 postulated that “robotic-assisted proctectomy with IPAA can be performed with comparable short-term clinical outcomes to laparoscopy but is associated with a longer duration of surgery and higher surgery costs.” Those authors optimistically stated further that “as experience increases, some advantages may become evident regarding operative time, postoperative recovery, and LOS; the robotic procedure might then become cost-efficient.” Another team of investigators compared the robotic approach with proctectomy and IPAA, finding that, in their operating suite, it offered significant technical advantages to the laparoscopic approach, expanding minimally invasive surgical techniques to IPAA [9]. Reporting a year later, after considerable experience with the robotic versus conventional IPAA, the same team concluded that robotic-assisted IPAA was equivalent to laparoscopic IPAA except for significantly higher costs [19].

Previous case-matched analysis from a high-volume center demonstrated that the robotic-assisted procedure reduced the likelihood of conversion to open surgery, decreased intraoperative blood loss, and shortened hospitalization while maintaining comparable perioperative outcomes [20]. However, these potential advantages were not corroborated in the present nationwide analysis, which demonstrated no significant difference in short-term clinical outcomes between robotic and laparoscopic approaches. Results of other studies, although limited by single-center case experience, still agreed to some extent on the cost issue associated with the robotic-assisted IPAA procedure and its modest clinical advantages [11, 21]. In a study of acute severe UC (ASUC), Lauricella et al. [22] found that robotic versus laparoscopic IPAA showed similar results for safety and postoperative morbidity. Those authors also reported that the transanal approach (Ta-IPAA) showed comparable short- and medium-term functional results. Another team of investigators reported that robotic-assisted stapled-IPAA for patients with UC led to better short-term outcomes and preservation of defecatory function with lower anastomosis than conventional laparoscopic IPAA, suggesting the clinical advantages of the robotic approach in this field [23]. In a single-center feasibility study and technical description [24], the investigators combined staged laparoscopic colectomy with robotic completion proctectomy and conventional IPAA, reporting improved clinical and cosmetic outcomes. Although robotic-assisted IPAA offers enhanced visualization and control during pelvic dissection, its potential benefits may lie in long-term functional outcomes, such as improved pouch function or continence, which were not available in this database [25, 26]. As robotic surgery is more expensive, future research should clarify whether its potential advantages such as functional outcomes outweigh the additional costs [27, 28].

The absence of measurable short-term advantages in this study may partly reflect the early phase of robotic implementation during the study period. Certain additional robotics-associated factors, such as the steep learning curve of surgeons, have been noted by investigators in other previous studies evaluating robotic-assisted surgeries. A systematic review of learning curves in robot-assisted surgery found that variability in surgeon experience with robotic techniques, including the impact of the learning curve, may influence results, potentially increasing complications and operative times in early adopters [29]. Wong et al. [30] addressed the learning curve and offered several recommendations to offset or balance it, including prior surgical experience in the particular conventional surgery (e.g., IPAA in the present report), operating within a mature robotic surgical unit with typical case complexity, robotic surgical simulation training beforehand, spending time as a bedside first assistant, and completing a structured training program with proctorship, if available. More recently, Violante et al. [31] challenged the traditional notion of a fixed learning curve by demonstrating that surgeon proficiency in robotic IPAA follows an individualized learning trajectory rather than a standardized case-volume threshold, emphasizing the importance of tailored mentorship and cumulative procedural experience. In the present study, no effects of the learning curve were noted, perhaps because the focus was on short-term outcomes, for which results suggest that outcomes may converge between robotic and laparoscopic IPAA when performed by skilled surgeons, as robotic advantages in dexterity and visualization may not provide additional benefits in uncomplicated cases.

Lastly, the 2016–2020 period analyzed in this study reflects an earlier stage in the learning curve for most robotic colorectal surgeons. Therefore, a follow-up analysis with more contemporary data may provide additional insights as surgical experience and technology continue to evolve. Furthermore, this study highlights the need for prospective, randomized trials to validate these findings and to address remaining gaps, such as detailed operative metrics and long-term, patient-centered outcomes.

### Strengths and limitations

The present study presents results gained through using comprehensive and nationally representative data generalizable across the entire US population. This broad scope offers a distinct advantage over the more limited single-center studies, which may not capture the diversity of the wider patient population. However, several limitations should be acknowledged. Although PSM was applied to reduce confounding, the retrospective design of this study remains prone to selection bias. In particular, unmeasured confounding may persist, as surgeons may preferentially select the robotic approach for technically challenging cases (e.g., male sex, high BMI), a factor not fully captured in the NRD. Differences in outcomes across surgical approaches may reflect variability in surgeon expertise, institutional factors, or disease severity rather than the surgical platform itself. Furthermore, the relatively small sample size, particularly within the proctocolectomy subgroup, limited the feasibility of performing separate, adequately powered matching analyses for each procedure type. The database lacks key variables that could influence outcomes, such as the presence of a diverting ileostomy, the learning curve, surgeon experience, intraoperative details, and surgeon experience. Institutional factors, including variations in postoperative care pathways (enhanced recovery versus standard), surgical approach, and hospital type, were unavailable and may have influenced results. Additionally, as an administrative dataset, the NRD is subject to coding errors and underreporting of complications, and it does not allow for assessment of long-term or functional outcomes, while rare events such as surgical infection and in-hospital mortality may lack adequate statistical power. Therefore, findings should be interpreted with caution, and future prospective studies with detailed clinical and functional parameters are warranted to validate these observations.

## Conclusions

This nationwide cohort study provides contemporary evidence on the comparative effectiveness of robotic-assisted and laparoscopic IPAA for ulcerative colitis. Robotic-assisted IPAA was associated with significantly higher hospital costs but comparable short-term outcomes, including complication rates, venous thromboembolism, length of stay, and readmission rates. These findings indicate that although robotic IPAA represents a technically feasible and safe minimally invasive approach, its adoption should be guided by careful cost–benefit considerations in the absence of clear short-term clinical advantages. Further prospective and longitudinal studies are warranted to clarify the potential long-term functional and quality-of-life benefits of robotic IPAA.

## Supplementary Information

Below is the link to the electronic supplementary material.

Supplementary file1 (DOCX 31 KB)

## Data Availability

All results supporting the findings of this study are included in the manuscript and its supplementary materials.
