# Supplementary material for: Robotic versus laparoscopic ileal pouch-anal anastomosis for ulcerative colitis: an analysis of the Nationwide readmission database, 2016–2020
Source: Tech Coloproctol. 2026 May 12;30(1):95. doi: 10.1007/s10151-025-03285-2 (PMC13337623; doi:10.1007/s10151-025-03285-2)
Supplement: Supplementary file 1 — Supplementary file1 (DOCX 31 KB) [file 10151_2025_3285_MOESM1_ESM.docx]

**Supplemental Table 1.** ICD codes used in the study.

|  | ICD 10 Code |
| --- | --- |
| Ulcerative colitis | **CM**: K51 |
| IPAA | **PCS**: 0D1B4ZQ, 0D1B8ZQ, 0D1B47Q, 0D1B4JQ, 0D1B4KQ, 0D1B8JQ, 0D1B87Q, 0D1B8KQ |
| Colon cancer | **CM**: C18 |
| Robotic surgery | **PCS**: 8E0W3CZ, 8E0W4CZ, 8E0W8CZ |
| Pneumonia | **CM**: J12-18 |
| Sepsis | **CM**: A40, A41, R65 |
| UTI | **CM**: N39.0, N30.00, N30.01, N30.10, N30.11, N30.20, N30.21, N30.22, N30.9, N39.1, N39.3, N39.4, N10, N11, N12, N13 |
| Surgical site infection | **CM**: T81.4XXA, T81.4XXD, T81.4XXS, O86.0, O86.1 |
| VTE | **CM**: I26.0, I26.9, I80.0-I80.3, I80.8, I80.9, I81, I82, O08.2, O22.3, O87.1, O88.2 |
| Respiratory failure/ mechanical ventilation | **CM**: J95.2-J95.8, J96.00, J96.90, J80, J81.0/ Z99.12  **PCS**: 5A1935Z, 5A1945Z, 5A1955Z |
| Acute kidney injury | **CM**: N17 |
| Shock | **CM**: R57, T81.1, T88.2, R65.21, A48.3 |
| Pouchitis, fistula, or mechanical issues with the pouch | **CM**: K91.85, K91.7 |
| Other unspecified postprocedural complications of digestive system | **CM**: K91.89 |
| Postprocedural intestinal obstruction | **CM**: K91.3 |
| Hemorrhage/hematoma/seroma | **CM**: K91.6, K91.87 |
| Proctocolectomy | **PCS**: 0DTE[0,4,7,8]ZZ, 0DBE[0,3,4,7,8]ZZ |
| Smoking | **CM**: Z71.6, Z72.0, Z86.43, Z87.891, F17, O99.33, T65.2 |
| Polyp of colon | **CM**: K63.5 |
| Obesity | **CM**: E66 |
| Diabetes | **CM**: E10-E13 |
| Chronic pulmonary disease | **CM**: I27.8, I27.9, J40 -J47, J4A, J68.4, J70.1, J70.3 |
| Chronic kidney disease | **CM**: I12.0, I13.1, N03.2-N03.7, N05.2-N05.7, N18, N19, N25.0, Z49.0-Z49.2, Z94.0, Z99.2 |
| Rheumatic disease | **CM**: M05.x, M06.x, M31.5, M32.x-M34.x, M35.1, M35.3, M36.0 |
| Hypertension | **CM**: I10-I16, I1A |
| Ischemic heart disease/ heart failure | **CM**: I20-I25, I50 |
| Malnutrition/Protein-Calorie Malnutrition | **CM**: E40-E46 |
| Anemia | **CM**: D50-D53, D64 |
| Use of Immunosuppressive Therapy | **CM**: Z79.52 |

ICD, International Classification of Disease; CM, clinical modification; PCS, procedure coding system.

**Supplemental table 2. Characteristics of the study population.**

|  | **All patients**  (n=820) | **Robotic-assisted** | | ***p-value*** | ***SMD*** |
| --- | --- | --- | --- | --- | --- |
|  |  | Yes  (n=256) | No  (n=564) |  |  |
| **Demography** |  |  |  |  |  |
| **Age, years** | 38.9 ± 0.5 | 39.0 ± 0.9 | 38.9 ± 0.6 | 0.913 | 0.019 |
| 18-29 | 266 (31.7) | 89 (33.9) | 177 (30.7) | 0.650 | **0.118** |
| 30-39 | 188 (23.3) | 52 (20.5) | 136 (24.5) |  |  |
| 40-49 | 157 (19.1) | 51 (20.1) | 106 (18.7) |  |  |
| 50-59 | 123 (15.4) | 41 (16.0) | 82 (15.1) |  |  |
| 60+ | 86 (10.5) | 23 (9.5) | 63 (10.9) |  |  |
| **Sex** |  |  |  | **0.020** | **0.183** |
| Male | 469 (56.6) | 166 (62.9) | 303 (53.9) |  |  |
| Female | 351 (43.4) | 90 (37.1) | 261 (46.1) |  |  |
| **Procedure** |  |  |  | 0.096 | **0.161** |
| Proctocolectomy | 182 (23.6) | 46 (18.9) | 136 (25.6) |  |  |
| Proctectomy | 638 (76.4) | 210 (81.1) | 428 (74.4) |  |  |
| **Smoking** | 192 (23.1) | 69 (27.5) | 123 (21.1) | **0.041** | **0.148** |
| **Insurance status/primary payer** |  |  |  | 0.417 | 0.097 |
| Medicare/Medicaid | 163 (19.0) | 56 (19.5) | 107 (18.8) |  |  |
| Private including HMO | 622 (77.0) | 185 (75.2) | 437 (77.8) |  |  |
| Self-pay/no-charge/other | 34 (4.0) | 14 (5.3) | 20 (3.4) |  |  |
| Missing | 1 | 1 | 0 |  |  |
| **Major comorbidities** |  |  |  |  |  |
| Polyp of the colon | 4 (0.4) | 2 (0.5) | 2 (0.3) | 0.615 | 0.033 |
| Obesity | 58 (7.7) | 25 (10.6) | 33 (6.4) | 0.058 | **0.151** |
| Diabetes | 44 (6.2) | 15 (6.6) | 29 (6.0) | 0.800 | 0.022 |
| Chronic pulmonary disease | 68 (8.3) | 20 (7.8) | 48 (8.5) | 0.731 | 0.027 |
| Chronic kidney disease | 12 (1.6) | 4 (1.7) | 8 (1.6) | 0.923 | 0.008 |
| Rheumatic disease | 11 (1.4) | 5 (2.1) | 6 (1.1) | 0.318 | 0.081 |
| Hypertension | 118 (14.4) | 38 (14.8) | 80 (14.2) | 0.824 | 0.017 |
| Ischemic heart disease/ heart failure | 11 (1.7) | 2 (1.4) | 9 (1.9) | 0.760 | 0.036 |
| Malnutrition/Protein-Calorie Malnutrition | 60 (7.1) | 24 (9.4) | 36 (6.1) | 0.132 | **0.125** |
| Anemia | 143 (17.7) | 55 (22.4) | 88 (15.6) | **0.016** | **0.173** |
| Use of Immunosuppressive Therapy | 65 (8.4) | 17 (7.4) | 48 (8.9) | 0.493 | 0.055 |
| **CCI** |  |  |  | 0.896 | 0.066 |
| 0 | 656 (78.9) | 204 (79.4) | 452 (78.6) |  |  |
| 1 | 113 (13.8) | 34 (12.5) | 79 (14.3) |  |  |
| 2 | 28 (4.3) | 10 (4.7) | 18 (4.1) |  |  |
| 3+ | 23 (3.1) | 8 (3.5) | 15 (3.0) |  |  |
| **Admission type** |  |  |  | 0.177 | **0.104** |
| Elective | 786 (96.2) | 249 (97.6) | 537 (95.7) |  |  |
| Emergent | 34 (3.8) | 7 (2.4) | 27 (4.3) |  |  |
| **Weekend admission** | 7 (0.8) | 2 (0.8) | 5 (0.9) | 0.873 | 0.012 |
| **Year of admission** |  |  |  | **0.021** | **0.400** |
| 2016 | 157 (20.5) | 31 (14.2) | 126 (23.2) |  |  |
| 2017 | 208 (25.2) | 47 (18.4) | 161 (28.1) |  |  |
| 2018 | 161 (18.8) | 60 (22.2) | 101 (17.3) |  |  |
| 2019 | 156 (18.7) | 55 (21.6) | 101 (17.5) |  |  |
| 2020 | 138 (16.8) | 63 (23.6) | 75 (13.9) |  |  |
| **Hospital bed numbers** |  |  |  | **<0.001** | **0.368** |
| Small | 63 (9.7) | 11 (4.6) | 52 (11.9) |  |  |
| Medium | 169 (20.0) | 73 (28.3) | 96 (16.4) |  |  |
| Large | 588 (70.3) | 172 (67.1) | 416 (71.7) |  |  |

Abbreviations: CCI, Charlson’s comorbidity index.

Continuous variables are presented as mean ± SE; categorical variables are presented as unweighted counts (weighted percentage).

P-values < 0.05 are shown in bold.

SMD>=0.1 are shown in bold.

**Supplemental table 3. In-hospital outcomes of the study population.**

| **Outcomes** | **All patients** | **Robotic-assisted** | | ***p-value*** |
| --- | --- | --- | --- | --- |
|  | (n=820) | Yes  (n=256) | No  (n=564) |  |
| **In-hospital mortality** | 1 (0.2) | 0 | 1 (0.2) | **-** |
| **LOS ^a^** | 5.8 ± 0.2 | 6.0 ± 0.4 | 5.8 ± 0.2 | 0.678 |
| **Total hospital costs ^a, b^** | 102.8 ± 3.9 | 127.1 ± 5.8 | 92.3 ± 4.5 | **<0.001** |
| **Any complications** | 132 (16.0) | 46 (16.8) | 86 (15.7) | 0.697 |
| **Surgical complications** | 76 (8.6) | 29 (10.9) | 47 (7.6) | 0.122 |
| Surgical site infection | 8 (0.8) | 6 (2.0) | 2 (0.3) | **0.002** |
| Pouchitis, fistula, or mechanical issues with the pouch | 9 (0.9) | 5 (1.7) | 4 (0.6) | 0.110 |
| Other unspecified postprocedural complications of the digestive system | 31 (3.7) | 9 (4.2) | 22 (3.5) | 0.607 |
| Postprocedural intestinal obstruction | 27 (3.0) | 8 (2.7) | 19 (3.2) | 0.645 |
| Hemorrhage/hematoma/seroma | 8 (0.9) | 4 (1.3) | 4 (0.7) | 0.351 |
| **Medical complications** | 78 (10.0) | 25 (9.1) | 53 (10.3) | 0.610 |
| Pneumonia | 4 (0.4) | 2 (0.7) | 2 (0.4) | 0.480 |
| Sepsis | 22 (2.6) | 5 (1.7) | 17 (3.1) | 0.242 |
| UTI | 13 (1.8) | 4 (1.6) | 9 (1.9) | 0.767 |
| VTE | 22 (2.9) | 9 (3.5) | 13 (2.6) | 0.518 |
| Respiratory failure/mechanical ventilation | 5 (0.6) | 2 (0.7) | 3 (0.5) | 0.694 |
| AKI | 22 (3.3) | 5 (2.1) | 17 (3.9) | 0.283 |
| Shock | 8 (1.0) | 1 (0.4) | 7 (1.3) | 0.216 |
| **30-day unplanned readmission ^a^** | 186 (22.0) | 50 (20.2) | 136 (22.8) | 0.416 |
| **90-day unplanned readmission ^a^** | 264 (31.1) | 74 (28.8) | 190 (32.1) | 0.355 |

Abbreviations: LOS, length of stay; UTI, urinary tract infection; VTE, venous thromboembolism; AKI, acute kidney injury.

Continuous variables are presented as mean ± SE; categorical variables are presented as unweighted counts (weighted percentage).

P-values < 0.05 are shown in bold.

^a^ Excluding patients who died in hospitals.

^b^ Thousand USD.
